# Value of Diagnostic Tools in the Diagnosis of Osteomyelitis: Pilot Study to Establish an Osteomyelitis Score

**DOI:** 10.3390/jcm12093057

**Published:** 2023-04-23

**Authors:** Roslind K. Hackenberg, Fabio Schmitt-Sánchez, Christoph Endler, Verena Tischler, Jayagopi Surendar, Kristian Welle, Koroush Kabir, Frank A. Schildberg

**Affiliations:** 1 Department of Orthopedics and Trauma Surgery, University Hospital Bonn, 53127 Bonn, Germany; 2 Department of Hand, Plastic and Reconstructive Surgery, Burn Center, BG Trauma Center Ludwigshafen, University of Heidelberg, 67071 Ludwigshafen, Germany; 3 Department of Diagnostic and Interventional Radiology, University Hospital Bonn, 53127 Bonn, Germany; 4 Institute of Pathology, University Hospital Bonn, 53127 Bonn, Germany

**Keywords:** osteomyelitis, score, diagnostics, histology, MRI

## Abstract

Osteomyelitis (OM) remains one of the most feared complications in bone surgery and trauma. Its diagnosis remains a major challenge due to lack of guidelines. The aim of this study was to prospectively analyze the value of the most common and available diagnostic tools and to establish an OM score to derive treatment recommendations. All patients with suspected OM were included in a prospective pilot study. All patients underwent blood sampling for C-reactive protein and white blood cell count analysis. Magnetic resonance imaging (MRI), and microbiologic and histopathologic samples, were taken from representative sites of initial debridement. All patients were treated according to their OM test results and followed for at least one year. Subsequently, the value of individual or combined diagnostic tools was analyzed in patients with confirmed OM and in patients in whom OM was ruled out. Based on these findings, an OM score was developed that included MRI, microbiology, and histopathology. The score identified all control patients and all but one OM patient, resulting in a correct diagnosis of 93.3%, which was validated in a second independent larger cohort. This was the first study to analyze the value of the most commonly used tools to diagnose OM. The proposed OM score provides a simple scoring system to safely interpret test results with high accuracy.

## 1. Introduction

Osteomyelitis (OM) is an inflammation of the bone caused by infecting microorganisms. The origin can be hematogenous (blood-born spread of bacteria to a predominantly pre-injured bone) or contiguous after open fractures, preceding surgery, adjacent skin ulcer or pressure sore [1,2]. It can be acute with severe localized pain, swelling, erythema, elevated temperature, malaise, exceed to bacteremia, and pyrexia or chronic with only minor clinical symptoms of subtle pain, swelling, redness and temperature elevation. In chronic OM tenderness, soft tissue remodeling, and recurrent sinuses may be present. If not treated adequately, acute OM can become chronic, and chronic OM can stay subclinical for years with a possible acute exacerbation at any time. The discrimination between acute and chronic OM is the presence of dead bone in chronic OM due to destruction and remodeling of the bone [2].

A precise and determined diagnostics is crucial to adequately treat OM, especially in OM of the limbs, where it is localized most frequently with at least 73.8% in the lower and 17.7% in the upper limb [3]. In the treatment of OM, the question of limb salvage versus an inevitable amputation is always raised. Despite medical progress, including more and more elaborate reconstructive procedures and new antibiotics allowing to aim for limb salvage more frequently, the prevalence of OM has been increasing [3]. The diagnostic workup remains a challenge and is the starting point for any treatment.

Besides the assessment of clinical symptoms, which may be subtle and nondescript, plain X-rays of the limb and laboratory workup including white blood count (WBC) and C-reactive protein (CRP) are basic diagnostics. In the early phase, X-rays may be normal, present with unspecific osteopenia or delayed fracture healing, so that differential diagnosis cannot be ruled out. Additionally, an elevated WBC and CRP are not specific for OM and can be present after trauma and surgery and in any inflammatory circumstance of the body [1,4,5,6,7,8]. In addition, a normal WBC and CRP may not rule out an OM, especially in clinically inapparent and chronic cases. Advanced diagnostic workup, including magnetic resonance imaging (MRI) and obtaining histological and microbiological samples, is not performed uniformly, due to a lack of consensus and standardized treatment recommendations [9].

Until now, there has been a vast lack of knowledge about the possibilities of advanced diagnostics, their proper performance, interpretation, and informative value. An adequate diagnostic workup, however, is inevitable and the basis for any treatment and, specifically, limb salvage. Thus, the aim of this study was to prospectively evaluate the value of diagnostic means in the diagnosis of OM and to develop a grading system to facilitate and standardize the diagnosis of OM in the limbs.

## 2. Materials and Methods

In this prospective monocenter pilot study in a clinic of maximum medical care, all patients with a minimum age of 18 years and a suspected OM of the limbs were included between January 2020 and March 2021. OM was suspected based on medical history and clinical findings such as local swelling, pain, redness, hyperthermia, and wound healing disorders with a persistent wound or recurrence of a wound or fistula, as well as fever and shivering.

Patients with and without a history of trauma and/or surgery at the local site, as well as those with and without local implants, were included. Age, gender, localization of suspected OM, length of hospital stay, treatment, predisposing factors, and complications were recorded. Complications were death, recurrent or persistent soft tissue infection, OM, and wound healing disorders, as well as adverse events necessitating revision surgery or intervention, such as joint dislocation, implant dislocation, postoperative bleeding, pneumonia, urinary tract infection, acute renal failure necessitating dialysis, thromboembolism, and myocardial infarction.

After inclusion into the study, an MRI with contrast medium was performed and a blood sample was taken from each patient to analyze the WBC and CRP. The presence of an implant was no contraindication for an MRI. In case of an implant at the site of suspected OM, the MRI was performed as a metal artifact reduction sequence. A computed tomography (CT) scan instead of an MRI was only considered as sufficient if the CT scan already showed distinct signs of OM. Distinct signs for OM in the CT were destruction of the bone, osteolysis, and the formation of sequestrums. Typical signs for OM in the MRI were as follows: focal decrease in bone marrow signal intensity on T1-weighted images, focal increase in signal intensity in the bone on T2-weighted, fat-suppressed images, focal bone marrow enhancement on gadolinium-enhanced fat suppressed T1-weighted images suggesting bone marrow edema, and in advanced stage, intraosseous abscess with formation of reactive bone surrounding intramedullary pus, subperiosteal abscess, sinus tract, ulceration, and cortical erosion [10,11,12].

During the first surgical debridement, microbiologic and histopathologic samples, each from soft tissue and bone, were taken and assessed by senior microbiologists and pathologists. Intraoperatively collected tissue specimens were homogenized and plated on Columbia agar with 5% sheep blood, MacConkey agar, chocolate agar, and Sabouraud agar (Becton & Dickinson, Bergen County, NJ, USA). In addition, samples were also cultured in thioglycolate boullion (Becton & Dickinson, Bergen County, NJ, USA). For anaerobic cultures, Schaedler and kanamycin/vancomycin agar plates (Becton & Dickinson, Bergen County, NJ, USA) were used. All cultures were grown at 5% CO_2_ and 35 °C for at least 14 days. All histopathologic samples were preserved in formalin, processed as paraffin sections, and stained with hemotoxylin and eosin. All bone samples were decalcified. Inflammation was diagnosed when inflammatory cells, specifically macrophages and granulocytes, were present.

Based on the diagnostic findings, patients were classified as “osteomyelitis” (OM) or “control” (CO) and treated accordingly. Classification was performed blinded and independently by two senior orthopedic and trauma surgeons. In case of disagreement, the case was reviewed by a third orthopedic and trauma surgeon. Patients were classified as “OM” when showing distinct signs of OM in the MRI and either having an active or chronic inflammation in histopathologic bone samples or an isolate of a microbiological pathogen in a bone sample. Patients were also classified as “OM” when only having signs compatible with OM in the MRI, such as minimal or diffuse contrast medium enhancement in the bone marrow, but a proof of active or chronic inflammation in histopathological bone samples combined with an isolate of microbiological pathogens in bone samples. Patients were classified as “CO” when no pathogen was isolated in bone samples, there were no signs of inflammation in histopathological bone samples, and there was an absence of distinct signs of “OM” in the MRI, or only when “OM” could not be completely ruled out. In all other cases, a reevaluation of the pre-analysis was performed, and testing was repeated. All patients were followed-up with for at least one year. Based on further diagnostic findings and the course of treatment, the classification into OM and CO was reevaluated in every patient and, in case of initial misdiagnosis, corrected.

The WBC, CRP, MRI, microbiologic, and histopathologic results were analyzed regarding their diagnostic value to predict the presence of OM between patients with and without a subsequent OM.

Furthermore, an OM score was established concerning the probability of a present OM based on the diagnostic findings. To confirm this score, we retrospectively validated it in a second independent cohort of 55 patients. Patients’ diagnostic findings were blindly evaluated according to the OM score and the results were compared with the initial clinician’s diagnosis. Descriptive statistical analysis was performed using GraphPad Prism, Version 9 (GraphPad Software, San Diego, CA, USA). Significant differences between the groups were identified using the unpaired t-test in normally distributed variables and the Mann-Whitney U test in non-normally distributed variables. The level of significance was defined at *p* < 0.05.

All patients included were willing to participate in the study and gave their written informed consent. The study was approved by the local ethics committee of the University Hospital Bonn (local review board number 277/19) and performed in accordance with the ethical standards of the institutional and national research committees and the 1964 Helsinki declaration and its later amendments.

## 3. Results

### 3.1. Patient Cohort and Clinical Presentation

A total of 15 patients were included in this pilot study. Nine patients had an OM and were assigned to the OM group. In 6 patients an OM was ruled out; thus, these patients were assigned to the CO group. There was no significant difference in the demographic data between the groups as displayed in Table 1.

In both groups the gender ratio was equivalent with 2:1 in favor of men. Despite, on average, a longer length of hospital stay and higher number of surgeries in patients of the OM group, there was no significant difference between both groups, respectively (length of hospital stay: *p* = 0.387, number of surgeries: *p* = 0.199).

All patients had a history of trauma, of whom 6 had a history of polytrauma (OM group n = 3, control group n = 3). There was no significant difference of the ratio of mono vs. polytrauma between the two groups (*p* = 0.422). There was no idiopathic wound healing disorder or OM. Additionally, in all patients of both groups, at least one risk factor for soft tissue infection and/or OM, such as an open fracture, underlying peripheral artery disease (PAD), underlying immunosuppressive or inflammatory disease or treatment (cancer, chemotherapy, chronic inflammatory autoimmune disease), and perforating injury, could be identified as shown in Table 2.

Of the 9 OM patients, in 6 an acute OM and in 3 a chronic OM was diagnosed. Two patients with chronic OM refused radical bony debridement and bony reconstruction, and so their numbers of surgeries and lengths of hospital stay were comparably low. Excluding both these patients, neither was the length of hospital stay significantly longer (OM group: 101 ± 67 d, CO group: 53 ± 34 d; *p* = 0.141), nor was the number of surgeries significantly higher (OM group: 15 ± 8 surgeries, CO group: 7 ± 4 surgeries; *p* = 0.087). In total, the lower limb (upper limb n = 3, lower limb n = 12) and the lower leg were affected most often in both groups; see Table 1. The rate of complications was not significantly higher in the OM group (n = 6) than the CO group (n = 2), even including 1 death in the OM group (*p* = 0.465). There was found to be no initial misdiagnosis after a follow-up of at least one year, and so no initial diagnosis needed to be corrected.

### 3.2. WBC and CRP

On average, both the WBC (OM group: 9.2 ± 2.8 × 10^9^/L, CO group: 6.4 ± 2.0 × 10^9^/L) and CRP were higher in the OM group (OM group: 46 ± 65 mg/L; CO group: 19 ± 13 mg/L). Despite the average WBC being within the reference range (female: 3.6–10.5 × 10^9^/L; male: 3.9–10.2 × 10^9^/L) in both groups, it was significantly higher in the OM group (*p* = 0.046). In the OM group, 4 patients showed an elevated WBC (>10.5 × 10^9^/L). In the CO group 1 patient had a lowered WBC (<3.6–10.5 × 10^9^/L).

The average CRP was elevated and above the reference range of 0–3 mg/L in both groups, however, not being significantly different between the groups (*p* = 0.344). In the OM group, 1 patient showed a normal value of the CRP while all other patients had an elevated CRP in both groups.

### 3.3. MRI

In the CO group, 2 patients (33.3%) showed osseous alterations with contrast medium enhancement in the MRI disallowing an exclusion of an OM. The remaining 4 patients had no typical OM-like alterations of the bone. Thus, in the CO group there was no positive MRI for OM; however, in 2 cases, an OM could not be ruled out.

In 8 patients of the OM group an MRI was performed. In 1 patient only a CT was performed and classified as sufficient, since it already showed distinct signs of OM with bone destruction, osteolysis, and sequestrums. In 7 patients (87.5%) of the OM group, OM-typical alterations were found in the MRI and, thus, were classified as positive for OM diagnosis. One OM patient (12.5%) exhibited osseous alterations, however, inconclusive of being in the context of OM or postoperative or stress reaction. This one MRI was not negative for OM, but did not confirm it either. Table 3 presents the essential diagnostic findings of the imaging procedures.

### 3.4. Microbiology

During surgical debridement, samples of the bone and soft tissue were taken routinely. In the CO group, no patient had a detection of a microorganism in the bone samples. In 5 of the 6 CO patients, bacteria could be isolated in the soft tissue samples. In 4 of them a polymicrobial infection was present, only 1 patient had an isolation of one pathogen.

In the OM group, 8 patients (88.9%) had an isolation of at least one pathogen in the bone samples. In 1 patient (11.1%) a pathogen could be isolated only in the soft tissue despite the presence of OM. Five patients (55.6%) had a monomicrobial OM. Three patients (33.3%) had a polymicrobial OM, with 2 patients having 2 pathogens and 1 patient having 3 pathogens isolated. Four patients had additional pathogens isolated in the soft tissue and/or the course of treatment. No patient had an infection with a multi-resistant pathogen. Table 3 displays the isolated pathogens in the OM and CO groups.

### 3.5. Histopathology

Histopathologic samples of the bone and soft tissue were taken in the first debridement in all but 1 OM patient. In 1 OM patient only soft tissue samples were taken, which were positive for inflammation. A repetition was not performed since there was no revision surgery for persistent OM. In 2 CO patients, no additional histopathologic samples of the soft tissue were taken. In all patients of the OM group, the bone samples showed chronic granulating inflammations and, thus, were positive for OM (100%). In all patients of the OM group that had soft tissue samples taken (n = 8), an accompanying chronic granulating inflammation of the soft tissue was also diagnosed.

No patient in the CO group had signs of inflammation in the bone samples. Of the patients with additional soft tissue samples in the CO group, 3 had signs of chronic granulating inflammation in the soft tissue, and in 1 patient a peri-implant membrane formation was present. Based on the histopathologic bone samples, all patients were correctly diagnosed for having OM or ruling out an OM. Table 3 shows the histopathologic results of all patients.

### 3.6. Osteomyelitis Score

On the sole basis of histopathologic exams, the diagnosis could be made correctly in all cases. Due to the risk of implementation error in taking samples from non-representative areas resulting in false negative results, a combination of diagnostic tools is recommended. Thus, an OM score was established to strengthen the diagnosis.

The OM score includes MRI, microbiology, and histopathology. A CT scan will only be valid instead of an MRI if it already is distinct for OM. Due to a lack of informative value, both laboratory tests—WBC and CRP—were not included in the OM score. For each diagnostic procedure, a point score was assigned, then added up for each patient and interpreted according to Table 4.

By means of the OM score, the presence and absence of OM could be correctly diagnosed in 14 patients (93.3%) and treatment could be derived for all patients according to the score. Based on this OM score, in all patients in the CO group, an OM could be correctly ruled out (n = 6; 100%), as shown in Table 5. All OM patients but 1 could be correctly diagnosed as suffering from an OM. One patient was classified as indistinct. The reevaluation of the diagnostic tools showed that there were no histopathologic samples taken from bone, which could have led to a correct diagnosis of OM. However, despite not having proof of OM, that patient was classified as highly at risk and, OM being probable, was thus treated as having an acute OM.

**Figure 1:**
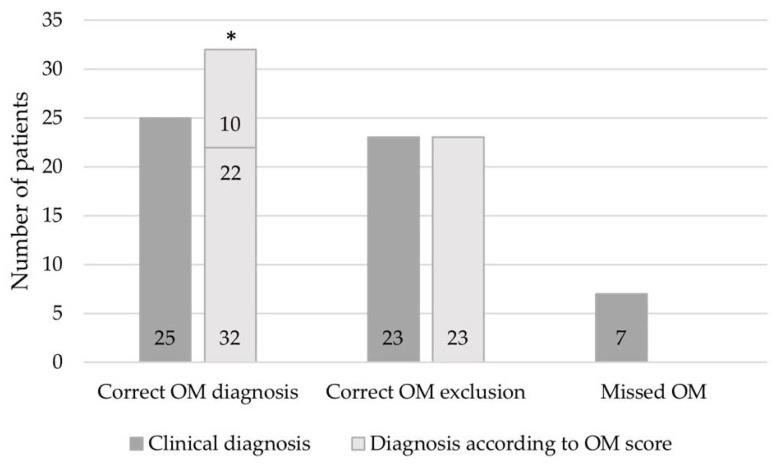
Validation of the OM score. A second independent cohort of 55 patients was used to validate the new OM score. Clinical diagnosis was compared to diagnosis according to OM score and the extent to which both modes correctly diagnosed, excluded, or misdiagnosed OM. * n = 32 patients: 22 patients with the correct diagnosis of OM (OM score of 2–3) and 10 patients with OM score of 1, recommending re-evaluation of diagnostic testing or treatment as OM.

To validate the new OM score, we utilized a second independent cohort of 55 patients (female: n = 19, 34.5%; male: n = 36, 65.5%) with a mean age of 59.8 ± 15.7 years. In this cohort, 25 patients (45.5%) were initially clinically diagnosed with OM. Using the OM score, 22 of these patients were also diagnosed with OM. The remaining 3 patients had an OM score of 1 with a recommendation to re-evaluate the test or treat as OM. Upon closer inspection, it was found that 2 patients did not receive complete histopathologic diagnostics, 2 did not have a properly interpreted MRI, and 1 had a false negative microbiologic result after antibiotic treatment. Most importantly, by clinical means alone, 30 patients were diagnosed as not having OM, of which 7 were misdiagnosed. Using the OM score, 23 patients were correctly diagnosed as not having OM and the 7 clinically missed OM were identified by the OM score as highly suspicious for OM (score of 1) with the recommendation to re-evaluate the test or treat as OM. Thus, by clinical means alone, only 87.3% of the patients were correctly diagnosed as having or not having OM and 7 patients with OM (12.7%) were missed (Figure 1). Using the OM score, all patients were correctly identified as having OM, not having OM, or being highly suspicious for OM. There were no false negative diagnoses using the OM score, and the 7 misdiagnosed patients would have been identified.

## 4. Discussion

Overall, OM is a rare disease with a prevalence of 16.7/100,000 inhabitants [3]. However, despite more and more modern treatment options, over a 10-year period from 2008 to 2018, its prevalence has increased by about 10.44% [3]. Its burden on the patient and health care providers, therefore, remains immense. A precise diagnostic is inevitable but crucial. Diagnostic tools such as clinical symptoms and blood tests are unspecific and may only aid as additional criteria. The correct administration and application of further diagnostic tools, specifically MRI, microbiological and histopathological tests, and their precise interpretation, is essential to a proper diagnosis of OM.

As soon as the diagnosis of an OM is set, a treatment consequence is inevitable. The initial diagnosis in early and acute stages may not be difficult to make, and may be correctly made in up to 80% of cases [13]. However, difficulties are specifically encountered in recurrent or persistent OM. Their therapy is crucial and often necessitates vast debridements and resections of bone segments, as well as implant removal in the case of present implants. This often results in the need for segmental bone reconstruction, not infrequently accompanying soft tissue reconstruction. If limb salvage is not possible, the only alternative is an amputation. The encounter with these severe therapeutical consequences may influence the correct interpretation of diagnostic findings and decision making. The honest interpretation of the diagnostic means should not be biased by a clinician’s concern regarding the challenging treatment options.

In this context, this is the first prospective study to evaluate the value of the most common diagnostic means for the diagnosis of OM in the limbs. Moreover, the established OM score offers a simple scoring system by which test results can be safely and accurately interpreted. There was no misdiagnosis based on the score. Only one OM patient was diagnosed as indistinct, where OM could not be safely diagnosed; however, treatment was recommended and conducted as having OM. Therefore, there was no missed OM and no overtreatment, and so the predictive value and accuracy of the score were high. The strengths and advantages of the proposed score are that it is easy to assess and that only those diagnostic tools have been included that are ubiquitously available and, thus, feasible for any surgeon.

Clinical symptoms are often subtle so that only 1–3 of the 5 typical signs of infection (pain, swelling, elevated temperature, redness, and loss of function) may be present [14,15]. While WBC is a good marker for sepsis, it has a low value in non-systemic infections and is largely neglected there [7,8,16,17]. CRP levels may be influenced by age, gender, weight, blood pressure [18], liver diseases [19], medications [20], and genetic preconditions [18,21,22]. Besides being elevated in case of infections [23], it may increase in any inflammatory condition such as rheumatoid arthritis, cardiovascular diseases, trauma, and cancer [4,5,6]. Both WBC and CRP are unspecific, and may be normal despite an OM or elevated without OM, as seen in the presented study population. Clinical symptoms as well as the CRP and WBC may aid in diagnosing OM but are unreliable diagnostic tools and, therefore, were not included in the proposed OM score.

Until today, microbiology has remained the gold standard in diagnosing infections. In bone and joint infections, however, it is known that microbial pathogens may only be isolated in 90% of acute and 51.4% of chronic OM, resulting in a high amount of false negative results [24]. In false negative cases, samples may not have been taken from representative regions of the affected bone, administered antibiotic treatment may have resulted in insufficient diagnostic power, or standardized culture mediums may have failed to isolate small colony variants [25,26,27]. In the presented study population, microbiologic organisms could also be isolated in only 88.9% of the patients. Hence, the sole diagnosis and consecutive treatment of OM should not be based on microbiology alone but rather on a combination of several diagnostics.

Despite the accuracy of 100% of the histopathology in the current study, a combination of diagnostic tools is still suggested. The risk of samples being taken from non-representative areas of the bone, resulting in false negative results, would have crucial consequences. Histopathological examinations, on the other hand, remain critical when taken from the representative area and can distinguish between acute, chronic, and acute on chronic OM even when microbial pathogens cannot be isolated [24].

In the presented study, performing an MRI belonged to the standard protocol for diagnosing OM. Still, MRI is not uniformly used—specifically, not when implants are present—even though several metal artifact-reducing sequences have well been introduced, and so, contraindications in the presence of orthopedic and trauma implants have become rare [28]. Due to its superiority in visualizing soft tissue contrasts compared to CT scans, it gains an accuracy of 71% to detect OM despite the presence of orthopedic implants and, thus, should always be favored or at least conducted additionally [29].

The advantage of the proposed score is its reduction of the diagnostic tools to the bare minimum and its clear instructions for treatment with strict cut-off levels. In the presented score, two diagnostic procedures may be sufficient if both are positive or negative; a third procedure, however, increases diagnostic safety. In individual cases, even one parameter may be enough to diagnose OM; however, in clinical practice, one parameter seems unsafe, and so a second should always be aimed for. Still, in all diagnostics, the inevitable pre-condition is that tissue samples are taken from representative areas, suggesting taking no less than 2 samples to reduce false negative results, and that the MRI is performed correctly, e.g., with contrast medium and metal artifact-reducing sequences when implants are present, prior to surgical bony debridement to rule out postoperative stress reactions of the bone, and preferably adjudged by a musculoskeletal radiologist.

As shown in the second retrospectively reviewed independent cohort, the new OM score could be validated without any misdiagnosis, while only 87.3% were correctly diagnosed by clinical means alone. Most importantly, even the 7 clinically missed OM would have been identified by the OM score. Contrary to the clinicians’ previous opinion, the score would have had a higher accuracy and the diagnosis of OM would have been more certain if the proposed OM score had been used.

In contrast to the proposed simple OM score, the 2011-introduced Osteomyelitis Diagnosis Score (ODS) to predict an OM contains 104 items and is quite demanding [13]. Furthermore, it contains broad windows with OM being “probable” (8–17 points) and “possible” (2–7 points) [13]. Therefore, until now, the score has not prevailed in everyday clinical practice despite its sensitivity of 82.8% and specificity of 95.8%. In a validation study [30], several items remained negative in all patients and, thus, seem redundant, and the score seems not practicable in everyday clinical practice.

Despite the first description of the score in this study, larger study populations will be needed to validate the score. However, since the score is simple, it may be possible to expand or modify certain parameters to further strengthen the score, e.g., by weighting items or adding subcategories such as the presence of metal implants. So far, however, the benefit of potential modifying factors and subcategories remains unclear, so the score is intended to be as simple and reliable as possible.

Due to the scarce overall incidence of OM and the prospective study design, the number of patients is low. Owing to this, the presented study does not claim to be confirmative but serves as a pilot study. Further studies with larger patient cohorts will be needed to verify the utility of the derived OM score.

## Figures and Tables

**Table 1:** Epidemiologic data.

		OM	CO		***p***
**Number of patients [n]**		9	6		-
Age [y] *		55 ± 19 [26; 79]	54 ± 18 [30; 78]		*p* = 0.910
**Gender [n]**	Female	3	2		*p* = 0.490
	Male	6	4		
Number of surgeries [n] *		13 ± 8 [2; 28]	7 ± 4 [3; 15]		*p* = 0.199
Length of hospital stay [d] *		81 ± 70 [6; 213]	53 ± 34 [5; 94]		*p* = 0.387
**Type of** **injury [n]**	Mono trauma	6	3		*p* = 0.422
	Polytrauma	3	3		
**Complications**	**Death [n]**	1	0		*p* = 0.465
	**Recurrent/persistent infection, OM, wound healing disorder, adverse events [n]**	5	2		
**Localization [n]**	Upper limb	Hand/finger	1	0	-
		Forearm/elbow	0	1	
		Upper arm/shoulder	1	0	
	Lower Limb	Ankle/foot	0	2	
		Lower leg/knee	5	3	
		Thigh/Hip	2	0	

* mean ± standard deviation [minimum; maximum].

**Table 2:** Injury entity and risk factors for soft tissue infection and/or OM.

Injury Entity and Risk Factors		OM	CO
Open fracture	Total	4	2
	With PAD * or immunosuppressive disease/treatment	0	0
Closed fracture	Total	4	3
	With multiple surgeries	1	0
	With PAD or immunosuppressive disease/treatment	1	3
	With multiple surgeries and PAD or immunosuppressive disease/treatment	2	0
Soft tissue/perforating injury without fracture	Total	1	1
	With PAD or immunosuppressive disease/treatment	0	1

* PAD: peripheral artery disease.

**Table 3:** Diagnostic findings in MRI, Microbiology and Histopathology.

Patient	Group	MRI	Microbiology	Histopathology
1	**OM**	**Positive** (Long-segment contrast medium enhancement in the bone as sign of OM)	**Bone**: *S. haemolyticus*	**Bone**: Chronic granulating inflammation
2	**OM**	**Positive** (Contrast medium enhancement in the bone adjacent to the fracture zone as signs of OM)	**Bone**: *S. epidermidis, Pseudomonas aeruginosa*	**Bone**: Active and chronic granulating inflammation in the medullary cavity
3	**OM**	**Positive** (Contrast medium enhancement in the bone with signs for distinct OM with abscess-forming soft tissue defect)	**Bone**: *S. aureus* (MSSA *)	**Bone**: Active and chronic granulating inflammation in the medullary cavity
4	**OM**	**Positive** (Contrast medium enhancement of the bone as a sign of OM)	**Bone**: *S. epidermidis*	**Bone**: Chronic granulating inflammation
7	**OM**	**Indistinct** (Slight contrast medium enhancement in the bone marrow of the without affection of the cortical bone, most likely to postoperative reaction; however, an inflammatory process (e.g., OM) cannot be ruled out)	**Bone**: *E. faecalis, Klebsiella pneumoniae, S. epidermidis*	**Bone**: Chronic active granulating inflammation
5	**OM**	**Positive** (Contrast medium enhancement in the bone and cortical bone defects with signs for OM and abscess-formation adjacent to a cortical bone defect)	**Bone**: *S. aureus* (MSSA *)	**Bone**: Chronic granulating inflammation
8	**OM**	**Positive** (Contrast medium enhancement of the bone with signs for OM)	**Bone**: *S. aureus* (MSSA *), *S. saccrolyticus*	**Bone**: Chronic granulating inflammation
6	**OM**	**CT: Positive** (Bony destruction with osteolysis and formation of bony sequestrums)	**Bone**: *S. capitis*	**Bone**: Chronic granulating inflammation
9	**OM**	**Positive** (Erosion of the cortical bone accompanied by signal alterations and contrast medium enhancement compatible with OM)	**Bone**: none **Soft tissue**: *Pseudomonas fluorescens*	**Soft tissue**: Moderate chronic granulating inflammation
10	**CO**	**Negative** (No OM-typical alterations with contrast medium enhancement only in the adjacent soft tissue)	**Bone**: none **Soft tissue**: *S. haemolyticus, Candida parapsilosis*	**Bone**: No signs for inflammation Soft tissue: Periprosthetic membrane, wear particle-induced type
11	**CO**	**Negative** (No contrast medium enhancement of the bone ruling out an inflammatory process of the bone)	**Bone**: none **Soft tissue**: *S. haemolyticus, S. epidermidis, Roseomonas mucosa*	**Bone**: No signs for inflammation **Soft tissue**: Chronic soft tissue inflammation
12	**CO**	**Indistinct** (Diffuse contrast medium enhancement of the bone compatible with stress reaction but also an inflammation, e.g., OM)	**Bone**: none **Soft tissue**: *E. cloacae, Acinetobacter bereziniae, Acinetobacter lwoffii*	**Bone**: No signs for inflammation and OM
13	**CO**	**Indistinct** (Diffuse contrast medium enhancement of the dorsal cortical bone compatible with an inflammatory process, such as OM, or stress reacion)	**Bone**: none **Soft tissue**: *S. aureus* (MSSA *), *S. agalactiae*	**Bone**: No signs for active inflammation **Soft tissue**: Chronic granulating active ulcerating inflammation with necrosis
14	**CO**	**Negative** (Minimal contrast medium enhancement of the superficial cortical bone; most likely postoperative reactive)	**Bone**: none **Soft tissue**: *S. epidermidis*	**Bone**: No signs for active granulating inflammation
15	**CO**	**Negative** (No contrast medium enhancement of the bone; no sign of OM)	**Bone**: none **Soft tissue**: none	**Bone**: No signs for chronic inflammation or OM **Soft tissue**: Connective tissue with focal purulent inflammation

* MSSA: methicillin-susceptible Staphylococcus aureus.

**Table 4:** Osteomyelitis Score.

Diagnostics		
Diagnostic Procedure	Points	Findings
MRI	+1 *	OM-typical alterations making OM very probable
	0	Unspecific osseous alterations with contrast medium enhancement or edema suggesting postoperative or posttraumatic stress reaction but not ruling out OM
	−1	No osseous alterations; absence of OM-typical bone reactions
Microbiology	+1	Isolation of pathogen in bone sample
	0	Isolation of pathogen only in soft tissue samples, but not in bone samples
	−1	No isolation of pathogen: neither in bone nor in soft tissue samples
Histopathology	+1	Granulating inflammation in bone samples
	0	Granulating inflammation in soft tissue samples, but not in bone samples
	−1	No granulating inflammation: neither in bone nor in tissue samples
**Interpretation**		
**Points**	**Interpretation**	**Treatment recommendation**
2–3	Presence of OM/OM most likely	Therapy of OM
1	Presence of OM indistinct	Reevaluate the adequate realization of the diagnostic tool (e.g., MRI performed with contrast medium, samples for microbiology and/or histopathology taken from representative sites?); high risk for developing an OM; thus, treat as OM
−3–0	Absence of OM/OM very unlikely	Therapy of underlying condition apart from OM, e.g., wound/soft tissue infection

* A CT with distinct signs for OM (destruction of the bone, osteolysis, and formation of sequestrums) may substitute an MRI and equivalently add +1 point to the score.

**Table 5:** Osteomyelitis score.

Group	Patient	MRI	Histo	Mibi	OM-Score
**OM**	1	1	1	1	3
	2	1	1	1	3
	3	1	1	1	3
	4	1	1	1	3
	5	0	1	1	2
	6	1	0 **	1	2
	7	1	1	1	3
	8	1 *	1	1	3
	9	1	0 **	0	1
**CO**	1	−1	0	0	−1
	2	−1	0	0	−1
	3	0	0 ***	0	0
	4	0	0	0	0
	5	−1	0 ***	0	−1
	6	−1	0	−1	−2

* Patient was only examined by CT. ** No bone sample was taken. *** No soft tissue sample was taken.

## Data Availability

Not applicable.
